# Possible glendonite mineral pseudomorphs in the aftermath of the end-Permian extinction

**DOI:** 10.1038/s41598-025-85443-y

**Published:** 2025-01-06

**Authors:** Musaab Al-Sarmi, Rachel Wood

**Affiliations:** Grant Institute, School of Geosciences, University of Edinburgh, James Hutton Road, Edinburgh, EH9 3FE UK

**Keywords:** Stratigraphy, Mineralogy, Sedimentology

## Abstract

Glendonites (from the precursor of ikaite, CaCO_3_.6H_2_O) preferentially precipitate within sediments in cold waters (− 2 to 7°C) via either organotrophic or methanogenic sulphate reduction. Here, we report the first occurrence of possible glendonites associated with the end Permian mass extinction in the earliest Triassic (ca. 252 Million years ago, Ma) subtropical marine carbonates on the Arabian Plateau, Oman. The authigenic carbonate crystals are small (< 2 mm) and precipitated either on bedding planes or reworked within micro cross-laminations, erosional scours, or lags at the base of calcisiltite turbidites, supporting a syn-depositional origin. The observed shape and macrostructure bear resemblance to that of glendonites. SEM and cathodoluminescent imaging reveals unzoned internal structures with three mineral phases: irregular, pseudo-hexagonal and spherical low-Mg calcite crystals (Type 1), low-Mg calcite cement (Type 2), and a later void-filling silica cement (Type 3). The pseudomorphs show δ^13^C values from − 0.14‰ to − 0.85‰ (mean − 0.43‰; n = 5) that are more positive than the associated micritic matrix, where values range from − 0.92‰ to − 2.39‰ (mean − 1.64‰; n = 7), indicating that oceanic dissolved inorganic carbon (DIC) was the primary carbon source rather than either methane or organic matter. These δ^13^C values significantly differ from typical δ^13^C signatures of authentic glendonites, except for Ordovician examples. If these are glendonites, we infer that they could have precipitated due to the unusually elevated alkalinity and pH (> 9) oceanic conditions present in the aftermath of the end-Permian extinction associated with highly disrupted carbon cycle dynamics, possibly accompanied with the upwelling of cold, anoxic oceanic water.

## Introduction

Glendonites, pseudomorphs after the precursor carbonate mineral ikaite (CaCO_3_.6H_2_O), have been found predominantly at high palaeolatitudes throughout the Phanerozoic and are commonly associated with cold-water deposits^1^. Ikaite precipitates in near-freezing temperatures due to its low solubility at 0°C^2,3^, and when stability conditions become unfavorable it decomposes and transforms into CaCO_3_ releasing water^4,5^. As a result, glendonites have traditionally been used as a cold-climate proxy indicating seawater temperatures from − 2 to 7°C^4^.

In marine sediments, two geochemical processes can induce ikaite precipitation: organotrophic sulphate reduction in which organic matter serves as a carbon source^4^, and methanogenic sulphate reduction where methane is anaerobically oxidised at the cost of sulphate^6^. Contrary to conventional findings mainly at high palaeolatitudes, glendonites have also been reported from greenhouse climatic episodes, from temperate and low palaeolatitudes^7–13^. The very low δ^13^C values of most marine glendonites and ikaite indicate a notable carbon acquisition from either organotrophic or methanogenic sulphate reduction. Both processes can lead to high carbonate, phosphate and/or sulphate concentrations, and elevated alkalinity, all which are essential for ikaite precipitations^2,14–17^. Experimental work has also shown that ikaite formation can be controlled by factors such as elevated pH and high concentrations of Mg^2+ 16^. Indeed, ikaite can precipitate in temperatures as high as 35°C when is pH > 9^18^. This raises questions as to whether glendonites in the geological record formed as a result of either cold spells^13^, or the upwelling of cold, nutrient-rich oceanic waters^7,8^, or cold bottom-water masses stemming from high latitudes^19^, or nucleation beyond their usual temperature stability range under unusual seawater chemistry states.

Here, we report small calcite aggregates that resemble glendonites in marine carbonate sediments located on the Arabian Plateau, Oman. These pseudomorphs are found in subtropical slope settings in the immediate aftermath of the end-Permian mass extinction, ca. 252 Ma, and so we further explore their potential relationship to the greatest known Phanerozoic biotic crisis.

### Geological setting

**Fig. 1:**
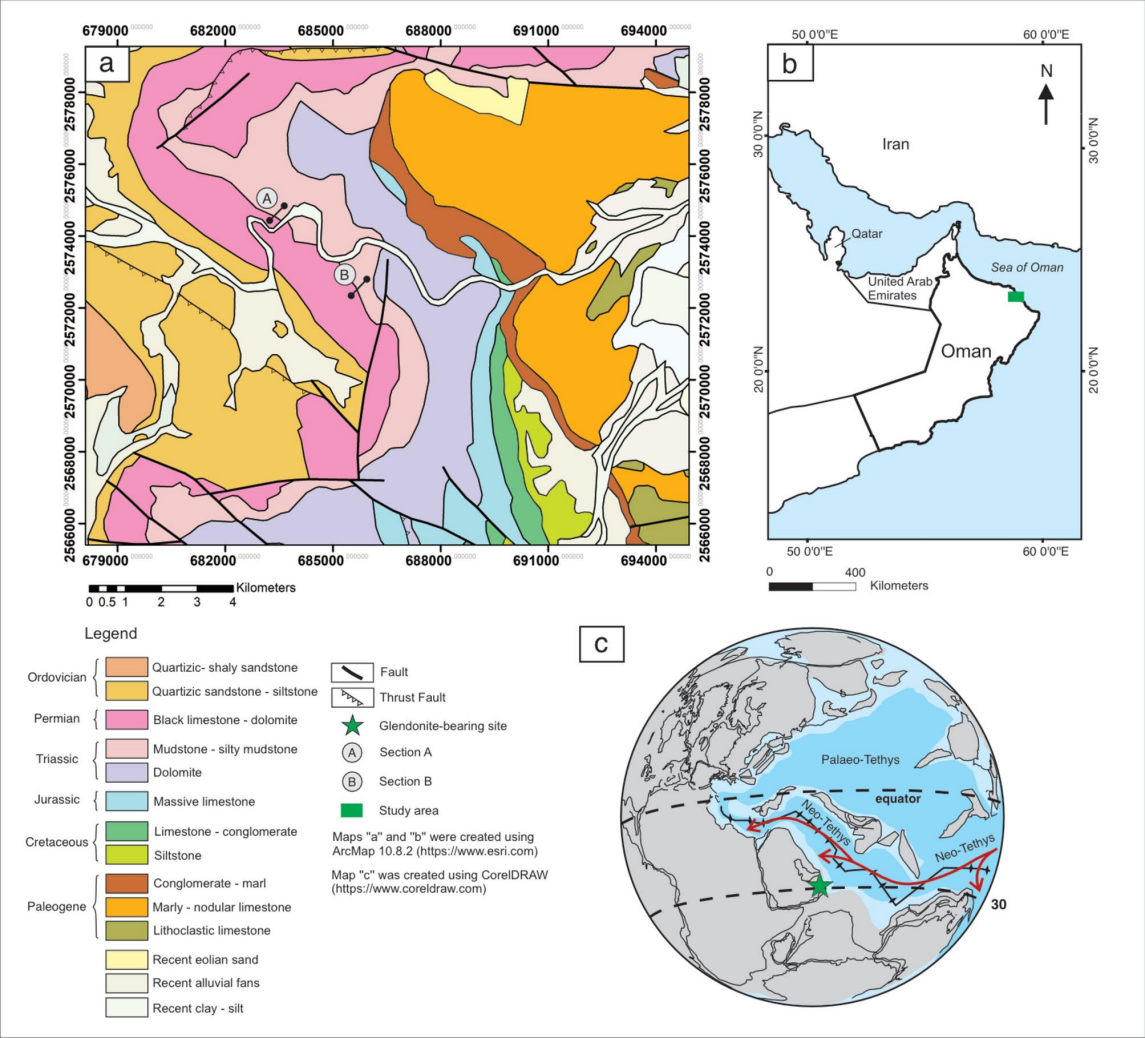
Geological setting of Early Triassic probable glendonites, Saiq Formation, Wadi Mijlas and nearby exposures in the Saih Hatat culmination, Oman. **a**: Geological map of study area modified after^20^, with the location of Sections (A, B). **b**: Geographical location. **c**: Paleogeographic map of the late Permian showing position of the study area and modelled upper ocean circulation^21^ modified after^22,23^.

Data are from two previously undescribed successions, Section A, ~ 66 m thick, and Section B, 30 m thick, found across the Permian–Triassic transition of the Saiq Formation in Wadi Mijlas and nearby exposures on the eastern flank of the Saih Hatat culmination, Oman (Figs. 1a,b). The successions are undeformed and laterally continuous for tens of kilometres. They consist mainly of marine carbonates deposited initially on mid to outer ramp, which evolved into mixed siliciclastic-carbonate on a slope depositional setting on the margin of an expansive carbonate platform that was connected to the central Neo-Tethyan Ocean (Fig. 1c).

Toward the late Changhsingian, the successions are characterised by m-scale shallowing-upward cycles within a Highstand Systems Tract, with mudstone/wackstone at the base passing into oolitic facies at the top of each cycle. These cycles often reveal normally graded beds with erosive bases inferred as tempestites and occasional hummocky cross-stratification. This then transitions to the Transgressive Systems Tract, organised into beds that are more normally graded and, in certain cases, display complete storm deposits, i.e. Dott-Bourgeous for typical storm deposits. The inferred PTB in both sections delineates an abrupt shift between two distinct lithologies. The interval above the inferred PTB revealed occasional matrix-supported intraformational breccia, likely debrite and cm-scaled deposits, depicting rhythmic patterns with occasional uni-direction micro-cross lamination and flute marks interpreted as turbidite-like deposits. Furthermore, the interval contains a slide-slumped unit that can be traced laterally for 200 m. All these features imply that these successions were likely deposited on a slope.

**Fig. 2:**
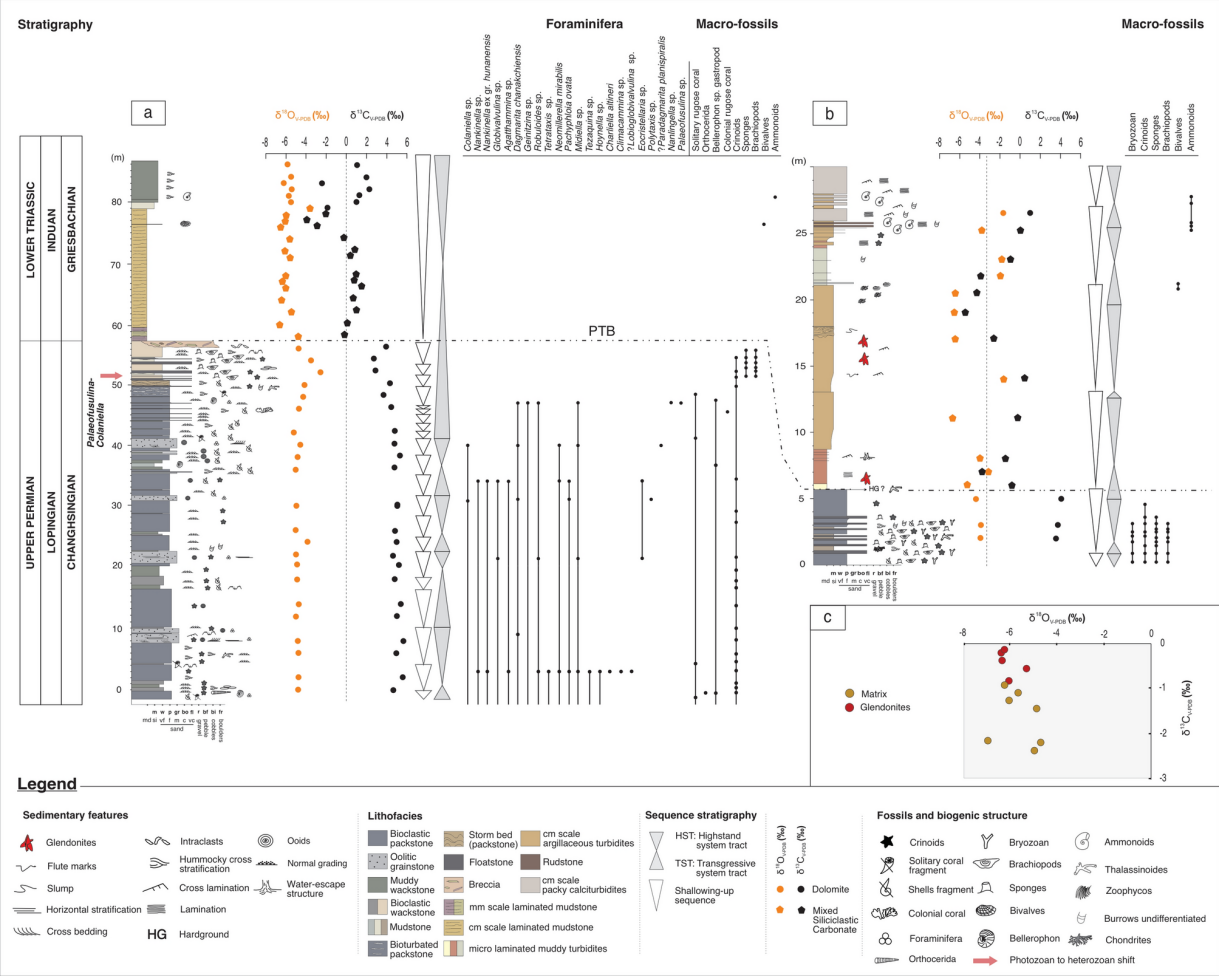
Measured sections along the Permian–Triassic (P-Tr) transition of the Saiq Formation, Wadi Mijlas and nearby exposures in the Saih Hatat culmination, Oman, showing lithostratigraphy, foraminiferal and macrofossil distribution, sequence stratigraphy, and δ^13^C chemostratigraphy of **a**: Section A, and **b**: Section B. The dashed-dotted line connecting Sections A and B represents the lateral correlation of the inferred PTB. **c**: Cross-plot of δ^13^C and δ^18^O values for pseudomorphs and matrix. A shift from photozoan to heterozoan in carbonate communities is observed below PTB in Section A (red arrow).

We have constrained the record in Section A stratigraphically by foraminiferal biostratigraphy and carbon isotope (δ^13^C) chemostratigraphy, which confirms the section to cover the *Paleofusulina-Colaniella* biozone of the latest Changhsingian (Permian) prior to a complete loss of biota coincident with a major negative δ^13^C excursion from 3.9‰ to − 0.2‰ at ~ 37 m, which is inferred to mark the Permian–Triassic boundary (PTB), before transitioning to the lowest Triassic (Fig. 2a; Supplement. Table S1). There is an abrupt shift in the ecology of the benthic communities from photozoan to heterozoan below the inferred PTB (Fig. 2a). In these heterozoan communities, identified brachiopods, including the latest Changhsingian, ?*Teserina nerii* (R. Posenato, pers. comm.), where its occurrence has been reported from the *Hindeodus changxingensis* Zone, see^24^ as well as *Leptodus richthofeni*.

Section B is the lateral equivalent of this section 5 km to the southeast and also shows a complete loss of biota and negative δ^13^C excursion from 4‰ to − 0.8‰ at 5.7 m (Fig. 2b; Supplement. Table S2). These follow the proposed PTB position in the Wadi Aday section of Saih Hatat^25^, but not the Quryat geological map^20^, where topmost members of the Saiq Formation, including the pseudomorphs-bearing strata (Sq_2b_), as well as Sq_3_-Sq_3_L are referred to as mudstone-silty mudstone (Fig. 1a), and considered to be of late Permian age. Two ammonoids are encountered at the top of Section B, possibly ?*Hypophiceras* aff. *H. gracile* and ?*Ophiceras connectens* (A. Baud, pers. comm.), revealing a Griesbachian age. Nevertheless, these forms cannot be conclusively identified, as homeomorphes of these ammonoids are also found later within the Smithian. However, their occurrence above the first negative carbon isotope increases the possibility that they are Griesbachian (A. Baud, pers. comm.). Three pseudomorph-bearing horizons are found above the inferred PTB in Section B, coincident with a significant transgression (Fig. 2b).

## Results

### Sedimentological and petrographic characteristics

The observed pseudomorphs in Section B are found in an interval that immediately follows the major negative δ^13^C excursion, in a remarkably distinct ~ 20 m thick transgressive unit of yellowish, cyclic, turbidite-like cm-scale calcisiltite beds that represent slope deposits. Most of the interval lacks fossil biota.

**Fig. 3:**
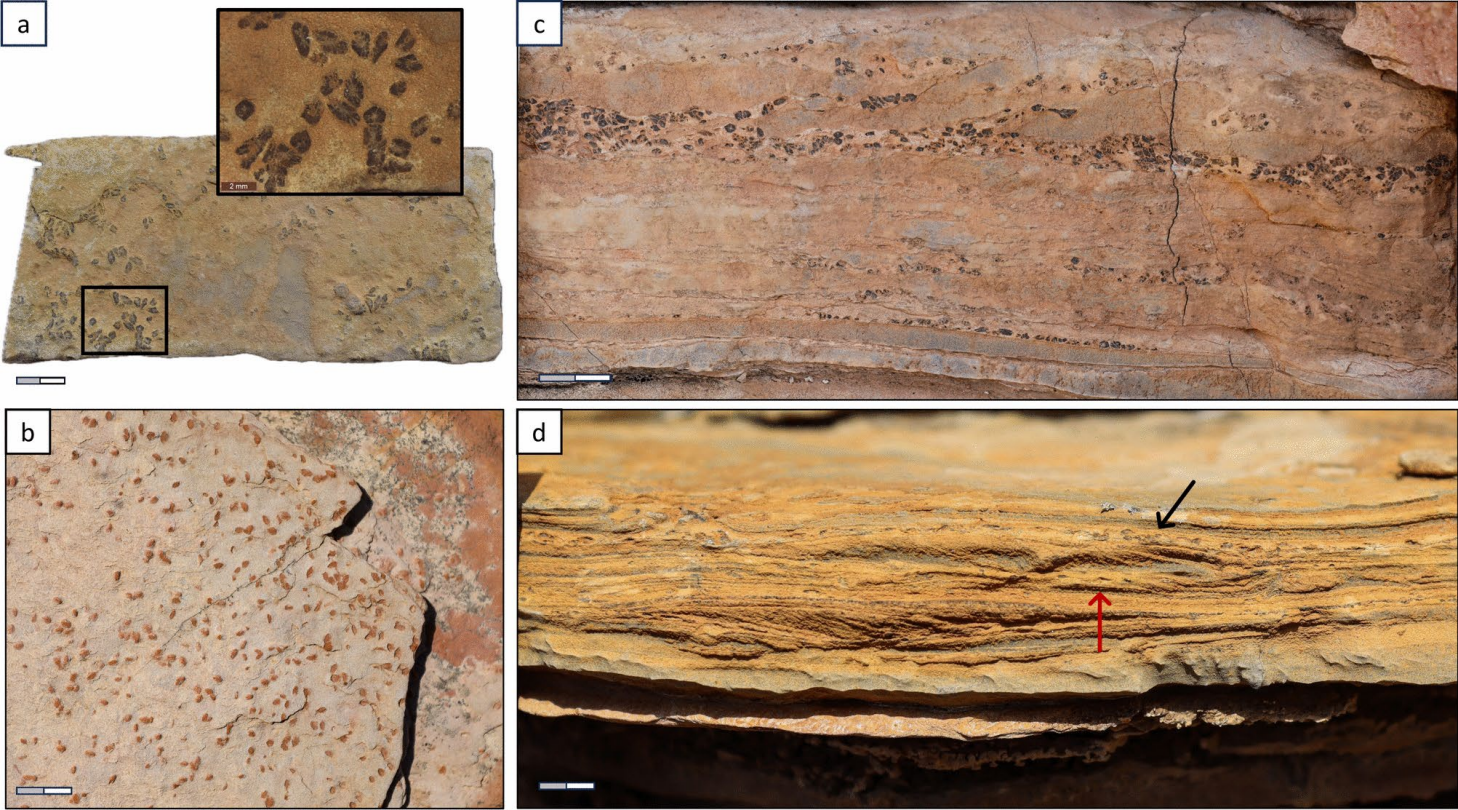
Pseudomorphs and their sedimentological habits, Early Triassic of the Saiq Formation, areas near Wadi Mijlas in the Saih Hatat culmination, Oman. **a (and inset)**: Small stellate-shaped glendonites clustered on a bedding plane (undefined level). **b**: Bedding plane with moulds of weathered glendonites (~ 6 m-level, outcrop section B). **c**: Clusters of pseudomorphs reworked and deposited as lags in erosional scours (17.2 m-level, outcrop section B). Note several sets of erosive surfaces associated with glendonite accumulation. **d**: Planar lamination and micro cross-lamination in calcisiltite turbidite beds, with pseudomorphs concentrated along planar laminations (black arrow), and reworked within micro-cross-laminae (red arrow) (15.75 m-level, outcrop section B). Scale bars = 1 cm.

The pseudomorphs occur at three stratigraphic horizons, at 0.7 m, 10 m, and 11.3 m above the inferred PTB (Fig. 2b). They were identified in three sedimentological habits: upon bedding planes of mirco-laminated marly (muddy) turbidites (Fig. 3a,b), reworked within erosive surfaces and scours (Fig.3c), and within micro-cross laminations of cm-scaled argillaceous turbidites (Fig. 3d).

**Fig. 4:**
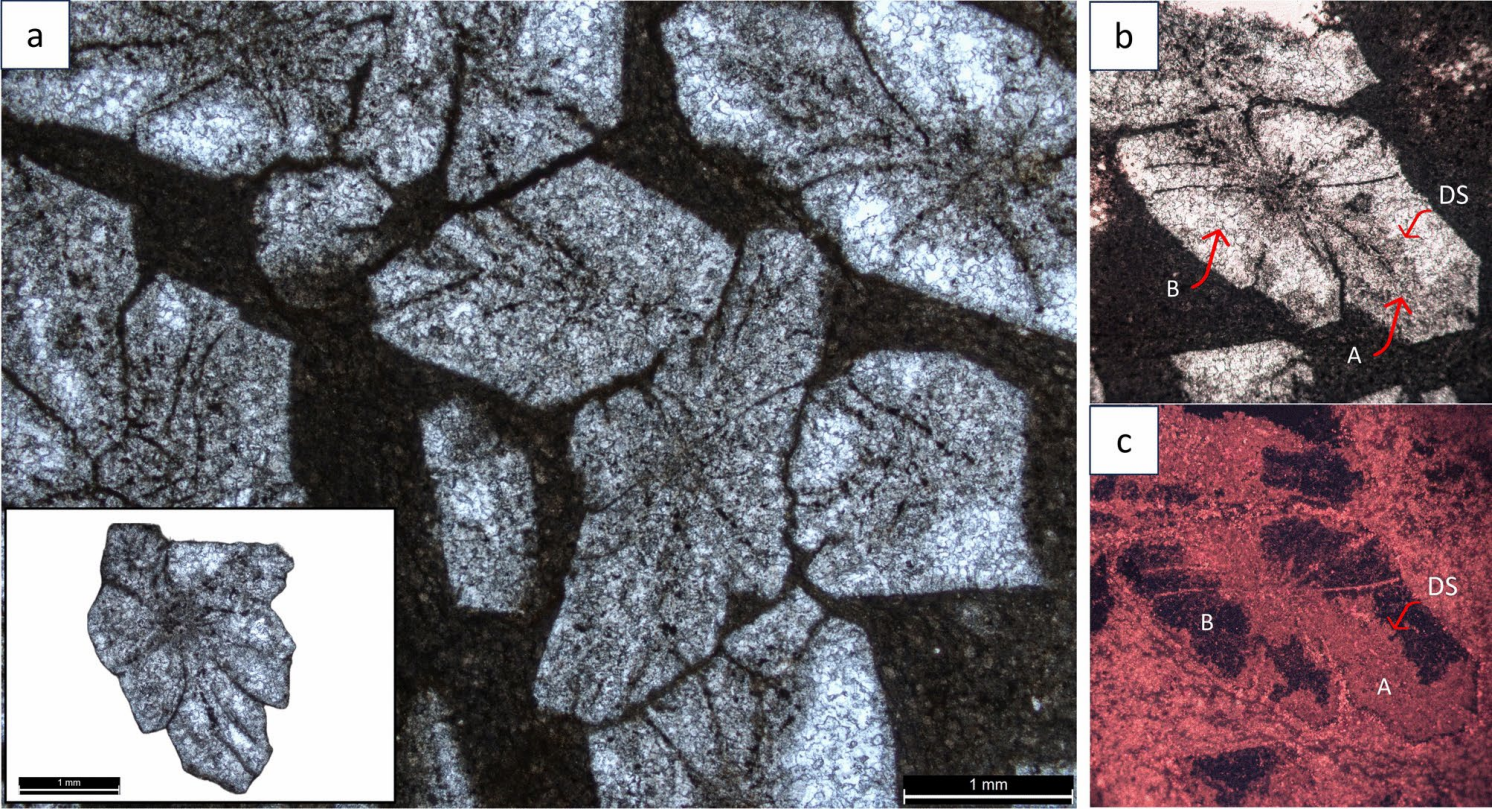
Photomicrographs of probable glendonites from the Early Triassic Saiq Formation near Wadi Mijlas, Oman. **a**: **(and inset)** Pseudomorphs with well-developed crystal faces enclosed by micritic matrix in plane polarised light (PPL). **b**: Primary ikaite-derived calcite (**A**) and clear drusy mosaic calcite (**B**) in PPL bounded by dissolution surface (DS). **c**: Cathodoluminescent (CL) image of **b** where (**A**) shows weak to dull luminescence, and (**B**) is non luminescent. DS = dissolution surface.

**Fig. 5:**
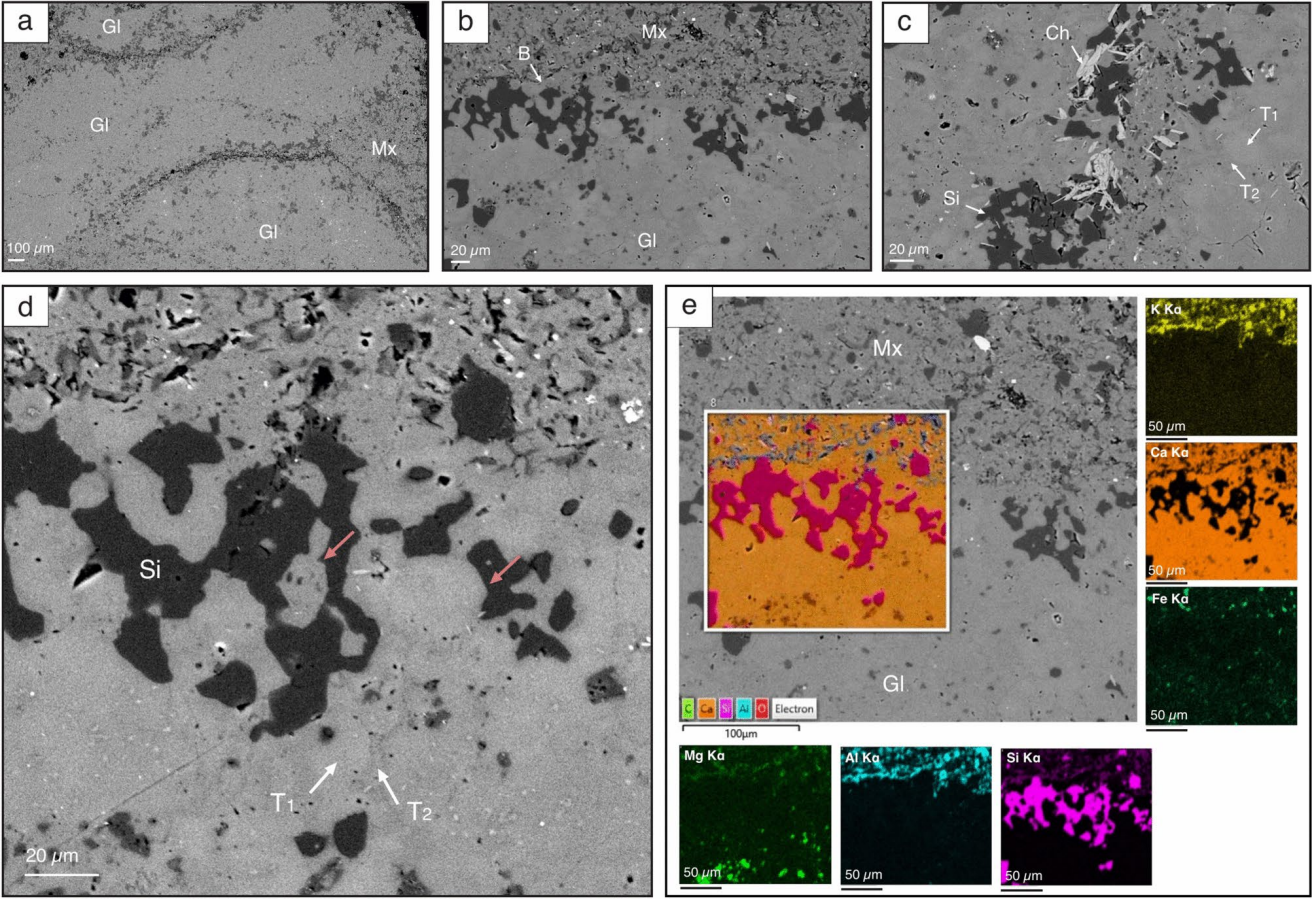
Back-scattered electron (BSE) images and scanning electron microscope energy-dispersive spectroscopy (SEM–EDS) elemental maps from the Early Triassic pseudomorphs, Saiq Formation near Wadi Mijlas, Oman. **a**: Overview of probable glendonites (Gl) and the surrounding matrix (Mx). **b**: A boundary (B) highlighting the difference between the matrix and the microgarnular to patchy texture in the pseudomorph crystal. **c**: Microgranular texture showing the three mineral phases: low-Mg calcite light grey cores Type 1 (T1), low-Mg darker grey calcite cement Type 2 (T2) and Type 3 pore-filling silica cement (Si). Note that the silica cement encloses bright elongated lithic chlorite (Ch) fragments, which are also found occasionally within the pseudomorph. **d**: Probable glendonite with the primary ikaite-derived calcite Type 1 (T1) and Type 2 (T2) as well as the Type 3 secondary silica cement (Si). Notice the pseudo-hexagonal crystals (red arrows). **e**: BSE image and SEM–EDS elemental maps of pseudomorph crystal and matrix. The packed, layered EDS map **(insert)** and elemental maps reveal that both ikaite-derived calcite Type 1 and Type 2 are made of calcite (orange) and are not enriched with Mg or Fe. The pseudomorph intercrystalline pore spaces are filled with silica cement (pink). The surrounding matrix shows enrichment in Al and K.

Pseudomorphs are small (1–2 mm) and display dark, grey-coloured stellate-shaped crystals, with well-defined crystal outlines that are embedded within a micritic matrix (Fig. 4a). Crystal aggregates show displacive growth between adjacent crystals or are interlocking (Fig. 4a). Some of these pseudomorphs typically exhibit bipyramidal shapes (Supplement. Fig. S1). The microscopic examination of pseudomorphs shows that the crystals are unzoned and homogenous throughout. Optical and cathodoluminescence imaging (CL) analysis reveals that pseudomorphs are made of inclusion-rich (probably organic matter) crystals that are decimicrometre-sized (~ 10–30 µm) and dull under CL (Fig. 4b,c). SEM backscattered electron (BSE) imaging reveals that the pseudomorphs are composed of minute calcite crystals with light grey cores ranging from 10 µm to 20 µm in size. These crystals are surrounded by a darker grey calcite cement (groundmass), with a patchy microgranular texture (Fig. 5). The calcite crystals are generally irregular, with some having pseudo-hexagonal and spherical habits (Fig. 5c,d; Supplement. Fig. S2). Energy dispersive spectroscopy (EDS) analysis indicates that both calcite cores and the calcite cement (groundmass) are low in Mg and Fe and exhibit no chemical variation (Fig. 5e; Supplement. Fig. S3f.,g). Intercrystalline pore spaces are filled with silica cement, and lithic fragments of chlorite were identified, trapped within the silica cement and, on occasion, within the pseudomorph calcite crystals (Fig. 5c). The matrix around the pseudomorph crystals exhibits elevated Al and K concentrations (Fig. 5e; Supplement. Fig. S3e), whereas pseudomorphs lack such elements (Fig. 5e). The pseudomorphs underwent dissolution, but the extent varies, resulting in irregular and enlarged pore spaces that were filled with secondary drusy void-filling non-luminescent centripetal spars (Fig. 4b,c).

### Stable isotopes

Pseudomorphs show δ^13^C values ranging from − 0.14‰ to − 0.85‰, with a mean =  − 0.43‰ (n = 5), and δ^18^O values ranging from − 5.32‰ to − 6.38‰, mean =  − 6.0‰ (n = 5). By contrast, the associated micritic matrix has δ^13^C values of − 0.92‰ to − 2.39‰, mean =  − 1.64‰ (n = 7), and δ^18^O values of − 4.73‰ to − 6.9‰, mean =  − 5.7‰ (n = 7). There is no correlation between δ^13^C and δ^18^O values (Fig. 2c). See data in Supplement Table S3.

## Discussion

The shape of these carbonate crystals is blocky, but they still form stellate clusters of blades with diamond-shaped tips and elongated bipyramidal blades typical for glendonites as documented from the geological record^1^ (Fig. 4a; Supplement. Fig. S1). It is possible that the pseudomorphs originated from gypsums and underwent complete replacement by calcite e.g.^26^. Gypsum can occur as a single crystal, intergrown twin complex or random clusters in a rosette-like aggregate known as desert roses^27^. However, it seems unlikely that these pseudomorphs originated from gypsum, given their occurrence within slope deposits. It is also implausible that re-deposited gypsum crystals in deeper settings would be the source, as gypsum is a soft mineral expected to disintegrate and, if so, be severely abraded from such long transportation.

The presence of pseudomorphs on bedding planes, along erosive surfaces and within cross-laminations suggest that these structures formed syn-depositionally and likely precipitated at the seafloor-water interface or perhaps slightly below. The lack of small-sized glendonites in the geological record may be attributable to sampling bias. Nevertheless, the mode of glendonite(?) occurrences described here remains unknown in other geological intervals. The mm-scale of these crystals compared to larger (cm-scaled) Paleozoic, Mesozoic, and Cenozoic examples (see Rogove et al.)^1^, suggests that conditions for nucleation and growth were brief and transient, and their occurrence at three stratigraphic horizons suggests that the facilitating, but transient, conditions re-occurred. We infer that the glendonites formed on or just under the sea floor during the very low energy, quiescent conditions between turbidite flows. These crystals then became entrained and re-worked in subsequent flows. The unzoned internal structures indicate rapid movement from the stability zone and complete transformation into calcite before the precipitation of diagenetic secondary calcite phases^28^. This suggests that these short-lived stability conditions were also quickly terminated. The resemblance of these pseudomorphs to ikaites/glendonites reported from the lagoon or semi-terrestrial settings is a noteworthy observation^29–33^. This implies that these pseudomorphs may have originated in shallower environments and then washed out into deeper settings. This seems, however, unlikely as the examined pseudomorphs depict well-formed, euhedral crystals embedded within micrite (Fig. 4a; Supplement. Fig. S1). Also, these small crystal aggregates show displacive growth between adjacent crystals or are interlocking (Fig. 4a) and show no preferred orientation along their long axis, revealing no signs of abrasion and transportation but rather indicating in situ growth (Supplement. Fig. S1). Nonetheless, this scenario cannot be completely disputed as evidence of reworked pseudomorphs has also been observed (Fig. 3c,d).

The ikaite-glendonite transformation causes ~ 70% volume loss in ikaite, leaving behind recrystallized calcite known as Type 1, which is then overgrown by the second calcite generation, known as Type 2; remaining pore spaces are filled with early burial drusy calcite, Type 3^28^. Only Type 1 and 2 represent the original ikaite-derived calcite^34^. Laboratory experiments have also shown that ikaite dehydrates and transforms into an admixture of vaterite and calcite^18,35^. Vaterite, being a polymorph of anhydrous CaCO_3_, can form spherical and hexagonal crystals^36^, but this polymorph is highly unstable, which causes it to transform into calcite^35,36^. The existence of pseudo-hexagonal and spherical cores within glendonites is an indication of former vaterite pseudomorphs that are later replaced by calcite^37^. The irregular, pseudo-hexagonal and spherical calcite crystals (light grey cores) distinguished here are likely ikaite-derived calcite and correspond to Type 1. The calcite cement surrounding these crystals resembles Type 2. This cement has low Mg content as Type 1. It also exhibits no Fe enrichment (Fig. 5e), indicating that it is more likely to originate from the low Mg ikaite precursor rather than from a later diagenetic origin. The pore-filling silica cement in glendonite intercrystalline pore spaces is referred to here as Type 3. Both petrography and δ^13^C and δ^18^O values indicate that these pseudomorphs have not undergone any significant overprinting by burial diagenesis.

Three possible sources of carbon are possible for glendonite formation: oceanic dissolved inorganic carbon (DIC) (δ^13^C values of 0–3‰), marine organic matter (δ^13^C values of − 17 to − 27‰), or methane (δ^13^C values of − 30 to − 110‰)^12^. Methane release from methanogenic and methane hydrates is proposed as one of the causes of the end of the Permian extinction event^38,39^, and glendonites could potentially archive such emissions^40–42^. δ^13^C values here, however, reveal no methanogenic signature. The stable isotope values of these pseudomorphs rather indicate that oceanic dissolved inorganic carbon (DIC) was the primary carbon source and do not support nucleation through either organotrophic or methanogenic sulphate reduction. However, it is possible that a small amount of carbon derived from organotrophic and methanogenic sulphate reductions may have contributed to a mixture of all carbon sources, although the DIC signature predominates. The δ^13^C and δ^18^O values are relatively similar to those derived from Ordovician (Tremadocian) glendonites^8,9^; however other glendonites in the geological record manifest much more negative values^1^. Measured δ^13^C for Antarctic ikaite (e.g. from the Brandfield Strait, the Firth of Tray and South Georgia)^5,43^ also indicates the presence of residual DIC.

The association of these pseudomorphs with the end-Permian mass extinction is notable. This marks a catastrophic event of rapid global warming rather than any cooling or significant influx of low temperature waters^44,45^. It is possible, however, that pseudomorph formation was aided by intermittent cold snaps during the earliest Triassic. However, the equatorial seawater temperatures estimated from δ^18^O_apatite_ of conodonts reveal a rapid temperature surge across the PTB, reaching a warming peak during the Griesbachian, followed by the first cooling event in the Dienerian^45^. Given the high temperature records during this time, it is unlikely that pseudomorph formation was a result of a cold snap. These pseudomorphs, may, however, be the product of cold and dense bottom water currents originating from high latitudes. Such currents might trigger the formation of ikaite in areas with otherwise warmer seawater temperatures. This scenario is perhaps unlikely as the Neo-Tethys Ocean is mainly influenced by a warm south equatorial current that moves from the east and spreads along the southern margin of Pangea (Fig. 1c). Furthermore, this proposed model would require strong temperature gradient in high latitudes and sea ice formation^19^, which would not be expected during the end Permian mass extinction. Indeed this period was characterized by intense global warming that extended into high palaeolatitudes^46,47^, with a minor temperature gradient between the equator and the poles that weakened thermohaline circulations^48^.

Glendonite formation could also have been facilitated by the local upwelling of cold, anoxic oceanic waters, as the pseudomorph-bearing strata coincided with a significant transgression and repeated anoxia has been proposed as the main cause of the extinction on the Arabian margin^49^. However, δ^18^O data do not record values that would support this, although there is insufficient evidence to confirm that these are primary signatures. Further, the palaeogeographic position of the studied locations was not on the western side of a continent, which might have experienced significant upwelling (Fig. 1c). Indeed extreme warming and ocean stratification^50^ would have weakened the global overturning circulation, resulting in a reduction in the upwelling processes of nutrient-rich water^51^. This had a significant impact on the prevailing upwelling system along the northwestern Pangean margin, which was stable and productive during the middle and late Permian but was largely terminated during the end of the Permian^52^. That the upwelling of cold, anoxic oceanic water masses acted as a trigger for ikaite growth cannot, however, be entirely ruled out. Evidence-based on variations in sulfur isotopes across the PTB suggests that upwelling of sulfidic deep ocean water masses indeed occurred at tropical latitudes in the eastern portion of the Palaeo-Tethys Ocean^53^. Additionally, the abrupt shift noted from photozoan to heterozoan communities below the inferred PTB (Fig. 2a) at a palaeo-subequatorial latitude may indicate an upwelling event of nutrients into shallow waters in the region^54^.

The presence of pseudomorphs is, however, consistent with locally elevated pH and alkalinity, which enables the precipitation of ikaite at warm, subtropical temperatures^18^. Further, these pseudomorphs contain silica. Silica precipitation is primarily influenced by changes in pH, as the solubility of silica rises significantly when pH levels increases beyond 9, resulting in precipitation when the pH level drops^55,56^. There is also abundant, independent evidence for increased alkalinity and pH in the immediate aftermath of the first extinction event in the form of ‘anomalous’ carbonate precipitations such as sea floor fans, large ooids, and microbialites e.g^57–59^, as well as elevated boron isotopic values in coeval successions from nearby UAE^60^. The rapid increase in weathering and detrital fluxes to the global ocean, seen in many sedimentary PTB records^61,62^, resulted in higher seawater alkalinity e.g.^63^, and prevailing anoxic to dysoxic sediment conditions during this period would also contribute to the elevation of bottom water alkalinity due to the bicarbonate input resulting from anaerobic degradation of organic matter^64,65^. The formation of pseudomorphs in the Saiq Formation, however, cannot be solely attributed to the elevated alkalinity. Modern ikaite has not been observed in warm areas, even in highly alkaline and P-rich water^37^. The conditions used for synthesising ikaite at a temperature beyond its stability^18^ are also not currently known in nature. Pseudomorph δ^13^C values rather imply that their formation was influenced by the oceanic dissolved inorganic reservoir, and their association with negative δ^13^C excursions and a mass extinction event suggest that both the pseudomorphs and rapid δ^13^C excursions were most likely caused by the unusual state of the oceanic dissolved inorganic carbon reservoir in the aftermath of the end-Permian mass extinction, possibly accompanied with the upwelling of cold, anoxic oceanic water.

## Conclusions

We present findings of Early Triassic authigenic carbonate pseudomorphs, from the Saiq Formation of the Arabian Plateau, Oman. The host sediment appears coincident with the end of the Permian mass extinction and associated negative carbon excursion, and their sedimentological occurrence confirms a syn-depositional origin. The shape and microfabric of these enigmatic carbonates are similar to glendonites (pseudomorphs after ikaite), although much smaller in size than previously studied. Bulk δ^13^C values demonstrate that their formation was unlikely to have been via either organotrophic or methanogenic sulphate reductions, but rather that DIC was the primary carbon source. Yet if these are glendonites, elevated pH and alkalinity probably facilitated their formation, possibly associated with upwelling of cold, anoxic oceanic water. We demonstrate, then, that glendonites can be associated with highly disrupted carbon cycle dynamics associated with mass extinctions, and controlled, at least in part, by dramatic changes in the inventory of oceanic dissolved inorganic carbon.

## Methods

We examined polished thin sections under an optical microscope and a Cathodoluminescence Cold Cathode CITL 8200 MK3A (CL) mounted on a Nikon optiphot microscope to identify differing luminescence^66^ at the Grant Institute, University of Edinburgh. A polished thin section of clustered pseudomorphs was coated with palladium and analysed using SEM backscattered electron (BSE) imaging and energy dispersive spectroscopy (EDS) at the Grant Institute, University of Edinburgh. The analysis was conducted with a Carl Zeiss SIGMA HD VP FEG (field-emission) SEM with Oxford Instruments AZtec EDS (and EBSD) analysis system at 20 kV and aperture size 30 um- standard.

Powders of sample size 0.08–1.0 mg were ground using a handheld micro-drill from hand samples. Between two and three glendonite crystals were drilled to create sufficient powder for a single analysis. These were analysed at the Iso Analytical laboratory, and were dissolved at 25 °C with 100% phosphoric acid, followed by conventional mass spectrometry using Continuous Flow-Isotope Ratio Mass Spectrometry (CF-IRMS), calibrated against the International Atomic Energy Agency (IAEA) standards, including NBS-18, NBS-19, and IAEA-CO-1. Results are reported as deviations from the VPDB standard (‰) and precision was measured at a level better than 0.1‰ for δ^13^C and δ^18^O (Supplement. Table S1-3).

## Supplementary Information

Supplementary Information.

## Data Availability

All data generated and analysed during this study have been included in this published article along with its Supplementary information file.
