# Supplementary material for: Possible glendonite mineral pseudomorphs in the aftermath of the end-Permian extinction
Source: Sci Rep. 2025 Jan 6;15:974. doi: 10.1038/s41598-025-85443-y (PMC11704205; doi:10.1038/s41598-025-85443-y)
Supplement: Supplementary file 1 — Supplementary Information. [file 41598_2025_85443_MOESM1_ESM.docx]

Possible glendonites in the aftermath of the end-Permian Extinction

**Musaab Al-Sarmi^1^* and Rachel Wood^1^**

**Supplementary Figure S1:** Early Triassic pseudomorphs from Saiq Formation, nearby Wadi Mijlas in the Saih Hatat culmination, Oman, displaying small stellate-shaped pseudomorphs clustered on a bedding plane; some crystals have elongated bipyramidal blades (arrows). Scale bar =1mm

**Supplementary Figure S2:** BSE image produced by SEM-EDS of early Triassic pseudomorphs from Saiq Formation, nearby Wadi Mijlas in the Saih Hatat culmination, Oman. These shows ikaite-derived calcite, with a preserved pseudohexagonal crystal of precursor vaterite^1^ (white arrow). The pore spaces are filled with silica cement (Si).

******

**Supplementary Figure S3:** BSE image (**a**) and **(b-g)** EDS point analysis measuring the elemental composition of the selected minerals within early Triassic pseudomorphs and their matrix from Saiq Formation, nearby Wadi Mijlas in the Saih Hatat culmination, Oman. (**b**) silica, (**c**-**d** & **f**-**g**) ikaite-derived calcite, and (**e**) elemental composition of the matrix.

**Supplementary Table S1:** Section A, the Permian-Triassic (P-Tr) transition of the Saiq Formation, Wadi Mijlas in the Saih Hatat culmination, Oman. Standard deviations for δ-^13^C and δ-^18^O are 0.05 and 0.06, respectively.

|  | **Amount measured** |  | **Result** δ**-^13^C_V-PDB_** | **Result** δ**-^18^O_V-PDB_** | **Corrected Result** δ**-^18^O_V-PDB_ for dolomite ^2^** |  |
| --- | --- | --- | --- | --- | --- | --- |
| **Lithology** | **(mg)** | **Height (m)** | **(‰)** | **(‰)** | **(‰)** | **Note** |
| Dolomite | 0.1204 | 0 | 5.18 | -4.9 | -4.94 | Bulk |
| Dolomite | 0.1081 | 2 | 4.57 | -5.01 | -5.06 | Bulk |
| Dolomite | 0.1056 | 4 | 4.99 | -3.86 | -3.90 | Bulk |
| Dolomite | 0.1145 | 6 | 4.79 | -5.02 | -5.07 | Bulk |
| Dolomite | 0.0995 | 10 | 5.03 | -4.94 | -4.99 | Bulk |
| Dolomite | 0.1272 | 16 | 4.76 | -5.03 | -5.08 | Bulk |
| Dolomite | 0.1176 | 18 | 5.3 | -4.84 | -4.89 | Bulk |
| Dolomite | 0.1155 | 20 | 4.76 | -4.6 | -4.64 | Bulk |
| Dolomite | 0.1198 | 22 | 4.79 | -5.22 | -5.27 | Bulk |
| Dolomite | 0.1046 | 26 | 4.432 | -4.723 | -4.77 | Bulk |
| Dolomite | 0.1056 | 28 | 3.67 | -4.29 | -4.34 | Bulk |
| Dolomite | 0.1072 | 30 | 4.29 | -4.15 | -4.19 | Bulk |
| Dolomite | 0.1182 | 32 | 2.86 | -2.57 | -2.60 | Bulk |
| Dolomite | 0.1224 | 34 | 2.7 | -3.51 | -3.55 | Bulk |
| Dolomite | 0.1173 | 36 | 3.93 | -4.74 | -4.79 | Bulk |
| Mixed siliciclastic carbonate | 0.1094 | 38 | -0.21 | -4.77 |  | Bulk |
| Mixed siliciclastic carbonate | 0.1267 | 40 | 0.06 | -6.62 |  | Bulk |
| Mixed siliciclastic carbonate | 0.1245 | 42 | 0.98 | -5.44 |  | Bulk |
| Mixed siliciclastic carbonate | 0.1105 | 44 | 0.64 | -6.41 |  | Bulk |
| Mixed siliciclastic carbonate | 0.1251 | 46 | 1.48 | -6.05 |  | Bulk |
| Mixed siliciclastic carbonate | 0.118 | 47 | 0.745 | -6.33 |  | Bulk |
| Mixed siliciclastic carbonate | 0.1121 | 48 | 0.96 | -5.97 |  | Bulk |
| Mixed siliciclastic carbonate | 0.0997 | 51 | 0.357 | -5.591 |  | Bulk |
| Mixed siliciclastic carbonate | 0.1261 | 52 | 0.83 | -6.07 |  | Bulk |
| Mixed siliciclastic carbonate | 0.103 | 54 | -0.27 | -5.63 |  | Bulk |
| Mixed siliciclastic carbonate | 0.129 | 56 | -2.93 | -6.52 |  | Bulk |
| Mixed siliciclastic carbonate | 0.1062 | 57 | -3.93 | -6.061 |  | Bulk |
| Mixed siliciclastic carbonate | 0.1214 | 58 | -2.04 | -5.97 |  | Bulk |
| Mixed siliciclastic carbonate | 0.0992 | 59 | -1.894 | -3.604 |  | Bulk |
| Dolomite | 0.1229 | 60 | 0.994 | -5.562 | -5.62 | Bulk |
| Dolomite | 0.125 | 61 | 1.25 | -5.73 | -5.79 | Bulk |
| Dolomite | 0.0973 | 62 | 2.24 | -5.47 | -5.52 | Bulk |
| Dolomite | 0.1226 | 63 | -2.43 | -6.24 | -6.30 | Bulk |
| Dolomite | 0.1245 | 64 | 1.97 | -5.507 | -5.56 | Bulk |
| Dolomite | 0.1059 | 66 | 1.05 | -5.85 | -5.91 | Bulk |

**Supplementary Table S2:** Section B, the Permian-Triassic (P-Tr) transition of the Saiq Formation, areas near Wadi Mijlas in the Saih Hatat culmination, Oman. Standard deviations for δ-^13^C and δ-^18^O are 0.075 and 0.06, respectively.

|  | **Amount measured** |  | **Result** δ**-^13^C_V-PDB_** | **Result** δ**-^18^O_V-PDB_** | **Corrected Result** δ**-^18^O_V-PDB_ for dolomite ^2^** |  |
| --- | --- | --- | --- | --- | --- | --- |
| **Lithology** | **(mg)** | **Height (m)** | **(‰)** | **(‰)** | **(‰)** | **Note** |
| Dolomite |  | 2 | 3.58 | -3.96 | -4.01 | Bulk |
| Dolomite |  | 3 | 3.76 | -3.91 | -3.96 | Bulk |
| Dolomite | 0.96 | 5 | 4.03 | -4.4 | -4.45 | Bulk |
| 35% carbonate | | 6 | -0.8 | -5.26 |  | Bulk |
| 15% carbonate | | 7 | -3.97 | -3.12 |  | Bulk |
| 15% carbonate | | 7 | -3.56 | -3.21 |  | repeated |
| Mixed siliciclastic carbonate | 1.06 | 8 | -1.41 | -4.22 |  | Bulk |
| Mixed siliciclastic carbonate | 0.98 | 8 | -1.51 | -3.83 |  | repeated |
| Mixed siliciclastic carbonate | 1.02 | 11 | -0.26 | -6.73 |  | Bulk |
| Mixed siliciclastic carbonate | 1.02 | 14 | 0.43 | -1.63 |  | Bulk |
| Mixed siliciclastic carbonate | 1 | 17 | -2.63 | -6.46 |  | Bulk |
| Mixed siliciclastic carbonate | 1.01 | 17 | -2.65 | -6.42 |  | repeated |
| 75% carbonate | | 19 | -5.45 | -6.55 |  | Bulk |
| 70% carbonate | | 20.5 | -4.29 | -6.44 |  | Bulk |
| 25% carbonate |  | 21.75 | -3.9 | -1.98 |  | Bulk |
| 45% carbonate | | 23 | -0.94 | -1.83 |  | Bulk |
| Limestone |  | 25.3 | 0.04 | -3.81 |  | Bulk |
| Dolomite |  | 26.5 | 1.01 | -1.68 | -1.70 | Bulk |

**Supplementary Table S3:** Stable isotope values from pseudomorphs from the Permian-Triassic (P-Tr) transition of the Saiq Formation, near Wadi Mijlas in the Saih Hatat culmination, Oman. Standard deviations for δ-^13^C and δ-^18^O are 0.03 and 0.07, respectively*.*

|  | **Amount measured** | **Result** δ**-^13^C_VPDB_** | **Result** δ**-^18^O_VPDB_** |  |
| --- | --- | --- | --- | --- |
| **Lithology** | **(mg)** | **(‰)** | **(‰)** | **Note** |
| pseudomorphs | 0.4 | -0.22 | -6.38 | Bulk |
| pseudomorphs | 0.2 | -0.14 | -6.26 | Bulk |
| pseudomorphs | 0.1 | -0.39 | -6.34 | Bulk |
| pseudomorphs | 0.08 | -0.85 | -6.06 | Bulk |
| pseudomorphs | 0.1 | -0.57 | -5.32 | Bulk |
| Matrix | 0.77 | -0.92 | -6.28 | Bulk |
| Matrix | 0.59 | -1.1 | -5.71 | Bulk |
| Matrix | 0.44 | -1.27 | -6.08 | Bulk |
| Matrix | 0.91 | -1.45 | -4.9 | Bulk |
| Matrix | 0.61 | -2.17 | -6.99 | Bulk |
| Matrix | 1.02 | -2.2 | -4.73 | Bulk |
| Matrix | 1.03 | -2.39 | -4.99 | Bulk |

**References**

1 Scheller, E. L., Grotzinger, J. & Ingalls, M. Guttulatic calcite: A carbonate microtexture that reveals frigid formation conditions. *Geology* **50**, 48-53 (2022). <https://doi.org/10.1130/G49312.1>

2 Rosenbaum, J. & Sheppard, S. An isotopic study of siderites, dolomites and ankerites at high temperatures. *Geochimica et cosmochimica acta* **50**, 1147-1150 (1986).
